# Chronic pain in transgender and gender-diverse youth: a biopsychosocial perspective

**DOI:** 10.3389/fpain.2025.1709268

**Published:** 2026-01-27

**Authors:** Gloria T. Han, Molly Basch, Diane Chen, Lonnie Zeltzer

**Affiliations:** 1 Departments of Anesthesiology and Pediatrics, Vanderbilt University Medical Center, Nashville, TN, United States; 2 Department of Endocrinology, Boston Children's Hospital, Boston, MA, United States; 3 Department of Psychiatry and Behavioral Sciences, Harvard Medical School, Boston, MA, United States; 4 Departments of Psychiatry and Behavioral Sciences, Pediatrics, and Medical Social Sciences, Northwestern University Feinberg School of Medicine, Chicago, IL, United States; 5 Pritzker Department of Psychiatry and Behavioral Health and Potocsnak Family Division of Adolescent & Young Adult Medicine, Ann & Robert H. Lurie Children's Hospital of Chicago, Chicago, IL, United States; 6 Departments of Pediatrics, Anesthesiology, and Psychiatry and Biobehavioral Sciences, University of California Los Angeles David Geffen School of Medicine, Los Angeles, CA, United States; 7 Creative Healing for Youth in Pain, Los Angeles, CA, United States

**Keywords:** biopsychosocial model, chronic pain, gender diverse adolescents, gender identity, minority stress, transgender, gender-affirming care

## Abstract

Chronic pain—including both chronic primary pain (e.g., headaches, widespread musculoskeletal pain, abdominal pain) and chronic secondary pain associated with other health conditions—represents a significant yet underrecognized health concern among transgender and gender-diverse (TGD) youth. While data on the prevalence of chronic pain in TGD youth remain limited, early studies indicate higher rates compared to their cisgender peers, highlighting the need for understanding factors underlying this co-occurrence. Chronic pain arises from a complex interplay of neurobiological, psychological, and social factors, and its heightened prevalence in TGD youth may be driven by the compounded impact of biopsychosocial stressors that disproportionately affect this group. This review summarizes neurobiological vulnerabilities, psychosocial factors, and societal and systemic barriers that may contribute to increased risk of chronic pain in TGD youth. We also examine the role of gender-affirming care in addressing these biopsychosocial vulnerabilities and explore its potential to alleviate some of the factors associated with chronic pain. Additionally, we identify critical gaps in the current body of research, such as the need for longitudinal studies and deeper exploration of the effects of medical interventions like pubertal suppression and exogenous hormones on chronic pain mechanisms and outcomes. By synthesizing the available evidence, we aim to guide future research and offer actionable recommendations to enhance clinical care and support for TGD youth experiencing chronic pain.

## Introduction

Pediatric chronic pain affects about one in five children and adolescents worldwide (20.8%) and is a significant public health concern, with headaches (25.7%), musculoskeletal pain (25.7%), multisite/general pain (21.0%), back pain (19.1%), and abdominal pain (17.3%) among the most common presentations (1). The impact of chronic pain during childhood extends far beyond the physical symptoms, often leading to functional limitations that disrupt daily life, including school attendance and academic achievement (2), peer relationships (3), and reduced participation in physical and social activities (4). These disruptions can hinder overall social, emotional, and physical development, leading to long-term consequences for quality of life (5). Furthermore, the economic burden of pediatric chronic pain is significant, with an estimated annual cost of $19.5 billion in the United States (6), encompassing both direct medical expenses and indirect costs such as parental work absenteeism and lost productivity. Families of children with chronic pain often experience significant financial strain, compounding the societal costs associated with this condition.

Sex differences in the prevalence of pediatric chronic pain are well-documented, with recent meta-analytic evidence for approximately 40% higher rates of chronic pain (especially headaches/migraines, musculoskeletal pain, and abdominal pain) in females compared to males (1, 7, 8). As most research to date has focused on presumed cisgender youth, there is a critical gap in understanding the prevalence and experience of chronic pain in non-cisgender or gender-diverse youth, who remain underacknowledged despite growing awareness of a potentially heightened burden of pain in this population (9–11). Gender-diverse is a term encompassing individuals whose gender identity and/or expression extends beyond the traditional male-female binary, including individuals who identify as transgender, gender queer, agender, and non-binary (12). While some transgender youth identify as gender-diverse, most identify as male or female but may not align with the sex they were assigned at birth. For the purposes of this review, we will focus on understanding the experience of chronic pain in transgender and gender-diverse (TGD) youth. We recognize that intersex individuals—those born with sex characteristics (e.g., chromosomes, gonads, hormones, or anatomy) that do not fit typical binary notions of male or female bodies—are also subject to medical pathologization, stigma, and health disparities (13–16). Their experiences may overlap with, but are distinct from, those of TGD youth. Empirical data on chronic pain in intersex youth are extremely limited, and a focused review is beyond the scope of this paper; however, we note the importance of including intersex populations in future work on gender diversity and pediatric pain.

The number of youth identifying as TGD is difficult to estimate; however, available reports indicate that 150,000 adolescents in the United States identify as transgender (17), and 9%–10% of 13- to 17-year-olds identify as TGD (18, 19). Younger generations are more likely to identify as transgender or non-binary, with Canadian census data showing that nearly two-thirds of this population is under the age of 35 (20). TGD youth face significant health disparities across physical and mental health domains (21). An overview of health outcomes of TGD youth by the Human Rights Campaign Foundation report (2023) indicates that TGD youth often face higher rates of harassment, bullying, social and peer rejection, and anxiety and depression, leading to increased stress and a greater likelihood of poor health outcomes, including chronic pain (22).

Although research on TGD youth and chronic pain is only beginning to emerge, initial studies focused on TGD adults suggest higher rates of migraines, musculoskeletal pain, and pelvic pain, with TGD adults almost twice as likely to report pain compared to cisgender adults (23). Similarly, recent pediatric studies have found that TGD youth present to pediatric pain clinics at disproportionately high rates (e.g., 34% in one clinical sample) (11), and results from a Swedish school-based survey study also indicated that 30% of TGD students in their study reported pain interfering with daily functioning, compared with 16% of cisgender girls and 8% of cisgender boys (24). These findings underscore the need to prioritize research in this area to inform clinical care that is appropriate for and tailored to this vulnerable group.

Importantly, chronic pain is a biopsychosocial phenomenon that emerges from the interplay between biological, psychological, and social factors (25). Biopsychosocial conceptualizations and models of care highlight the need to move beyond purely biomedical explanations and instead consider the multifaceted influences that shape pain experiences (26, 27). To this end, this review applies a biopsychosocial lens to summarize the neurobiological, psychosocial, and societal and systemic factors that may contribute to increased chronic pain prevalence in TGD youth. By integrating findings from the broader pediatric chronic pain literature with emerging insights into the experiences of TGD youth, we aim to highlight future research priorities and provide actionable recommendations for improving chronic pain prevention, management, and overall health outcomes in this underserved population. This narrative review is primarily intended for health professionals caring for youth with chronic pain (e.g., pediatric pain specialists, mental health clinicians, and providers involved in gender-affirming care), as well as researchers designing studies in this emerging area.

## Approach to the literature

In this review, we consider both chronic primary pain conditions, in which pain is the primary clinical problem, and chronic secondary pain, in which pain is attributed to an underlying health condition. Many of the mechanisms reviewed—such as central sensitization, minority stress, and barriers to gender-affirming care—are likely relevant across both groups; however, we anticipate that contextual challenges may differ (e.g., navigating pain in the context of other chronic illnesses). Where possible, we indicate when evidence or conceptual considerations apply more specifically to chronic primary vs. secondary pain. This article is a narrative, rather than systematic, review of the emerging literature on chronic pain in TGD youth. We identified relevant studies through targeted searches of databases (e.g., PubMed, PsycINFO, Google Scholar) using combinations of terms such as “transgender,” “gender diverse,” “nonbinary,” “adolescent,” “youth,” “chronic pain,” “pediatric pain,” and “somatic symptoms.” We also drew on reference lists of key articles and reviews and, when appropriate, included reports from major organizations to capture contemporary policy and advocacy contexts. Given the relatively small and rapidly evolving evidence base, our goal was to summarize and synthesize available data and to situate these findings within well-established pediatric pain and minority stress frameworks. Because we did not conduct a formal systematic search or risk-of-bias assessment, we may have missed some relevant studies, and the strength of evidence should be interpreted in light of these methodological limitations. As such, we summarize evidence for neurobiological, psychosocial, and societal and systemic contributors to chronic pain in TGD youth, and then discuss clinical implications and directions for future research.

## Evidence for biopsychosocial contributions to chronic pain in transgender and gender-diverse (TGD) youth

### Neurobiological factors

The onset of puberty is a critical period for the development of chronic pain, driven by hormonal, neurobiological, and psychosocial changes. Individuals assigned female at birth tend to enter puberty earlier than those assigned male at birth, exposing them to sex hormones such as estrogen and progesterone for a longer duration during adolescence. These hormones play a significant role in pain modulation and may contribute to the higher prevalence of chronic pain conditions observed in females (sex assigned at birth) compared to males (28–31). Estrogen, in particular, has been implicated in enhancing pain sensitivity through its effects on both the central and peripheral nervous systems, potentially leading to hyperalgesia (heightened pain sensitivity). Fluctuations in estrogen and progesterone levels throughout the menstrual cycle are associated with cyclical changes in pain intensity, as seen in conditions like menstrual migraines and premenstrual dysphoric disorder (32, 33). Progesterone, while less extensively studied, is thought to influence inflammatory and neuropathic pain (34, 35), with its effects varying based on individual hormonal profiles and the specific pain condition.

Neurobiological pain mechanisms are thought to contribute to sex differences in pain perception and processing, with females exhibiting less efficient descending inhibitory control—a key pain suppression system in the central nervous system (36, 37). Brain imaging studies have further identified heightened activation in brain regions responsible for pain proessing, such as the insula, anterior cingulate cortex, and thalamus, in females compared to males during experimental pain tasks (38–40). These findings suggest that hormonal influences, combined with structural and functional variations in pain-related neural pathways, contribute to sex-based differences in chronic pain vulnerability. For TGD individuals, the interplay between biological sex, gender identity, and gender-affirming medical interventions introduces additional complexities in understanding factors affecting the experience and maintenance of chronic pain.

Central sensitization, a neurobiological process in which the central nervous system (CNS) becomes hyper-responsive to pain stimuli, plays a crucial role in chronic pain vulnerability (41). This mechanism amplifies pain perception and prolongs pain experiences, even in the absence of ongoing tissue damage (42). Elevated rates of autism and other neurodevelopmental conditions have also been identified in TGD youth (43). Growing awareness of an overrepresentation of autistic youth presenting to pediatric chronic pain clinics (44) has concurrently stimulated an emerging area of work exploring this intersection. Preliminary studies indicate that altered sensory processing often seen in autism may lead to dysregulated pain modulation and increased prevalence of chronic pain (45–47). Autistic individuals may also experience heightened neural excitability and atypical connectivity in pain-related brain regions, raising the possibility that TGD youth—particularly those who are also autistic or have higher levels of autistic traits—may face unique neurobiological vulnerabilities to chronic pain. The overlap of chronic pain, autism, and TGD identity is an ongoing area of research (48) highlighting the importance of considering neurodevelopmental differences in future investigations of pain regulation in TGD youth.

### Psychosocial factors

Mental health comorbidities such as anxiety, depression, and post-traumatic stress disorder (PTSD) are disproportionately high among transgender youth (49) and are well-documented contributors to chronic pain (50, 51). Studies show that anxiety and depression amplify pain sensitivity through shared neural circuits involved in social and emotional distress and pain processing (52, 53). PTSD, which is more prevalent among TGD youth due to high rates of adverse childhood experiences, bullying, and social rejection, has been linked to somatic symptoms, including chronic pain (54, 55). Traumatic stress can also contribute to hyperarousal of the nervous system, heightening sensitivity to pain and increasing the risk for central sensitization of the nervous system and conditions involving chronic widespread pain (56, 57). For TGD youth, the intersection of mental health comorbidities and chronic pain often creates a vicious cycle in which pain exacerbates emotional distress, and emotional distress, in turn, magnifies or maintains pain.

One prominent framework for explaining higher rates of psychiatric comorbidities among TGD youth is the Minority Stress Model, which posits that individuals belonging to marginalized groups, such as TGD youth, experience chronic stress due to external factors like discrimination, stigma, bullying, social rejection, and family conflict, as well as internal factors (largely by-products of external ones) like identity concealment and internalized transphobia (58). Discrimination and stigma may occur across multiple settings, including schools, healthcare institutions, and peer relationships (59, 60). Studies consistently demonstrate that TGD youth are disproportionately targeted for peer victimization, bullying, and harassment compared to their cisgender peers (61). Additionally, social rejection and family conflict, including lack of parental acceptance, lead to social exclusion and isolation, intensifying exposure to minority stress and contributing to poor mental and physical health outcomes (62), including chronic pain. Indeed, a cross-sectional analysis of the 2020 and 2021 United States National Survey of Children's Health showed that TGD youth who had experienced discrimination were twice as likely to develop chronic pain (20.2%) compared to TGD youth who did not report discrimination (7.0%) (63).

The chronic stress associated with minority stress is not merely psychological; it profoundly impacts physiological systems that regulate pain perception. Stigma and harassment in schools—such as bullying and victimization—create environments of chronic stress, increasing the risk of emotional distress, physical trauma, and musculoskeletal pain due to physical altercations or prolonged muscle tension associated with stress (94). Dysregulation of the hypothalamic-pituitary-adrenal (HPA) axis, a central component of the body's stress response, is a well-documented consequence of prolonged stress exposure (64, 65). Repeated activation of the HPA axis leads to elevated cortisol levels, which can alter immune function and promote inflammatory processes. These changes contribute to peripheral and central sensitization, amplifying pain perception and prolonging pain experiences (41, 42).

TGD youth often experience gender dysphoria, defined as distress arising from incongruence between one's gender identity and assigned sex at birth. Gender dysphoria has been linked to increased bodily vigilance and hyper-awareness of physical sensations (95, 96), which can amplify pain perception (97). For TGD youth, routine interactions with their body—such as movement, clothing, and daily activities—may trigger distress, especially in invalidating social settings, leading to muscle tension, postural changes, and maladaptive coping mechanisms. The psychological burden of gender dysphoria can also result in social withdrawal and avoidance behaviors, such as reduced physical activity and poorer nutrition and hydration habits in efforts to avoid public restrooms (66). The negative impact of dysphoria-related distress on even basic health behaviors necessary for pain regulation and chronic pain management underscores the need for greater social and societal acceptance of TGD identities and youth access to gender-affirming approaches to care. These psychosocial stressors do not occur in isolation; they are embedded within family relationships, identity development, and broader social contexts that shape how TGD youth experience and respond to pain.

#### Parents, families, and visibility

Parents and caregivers play a central role in pediatric chronic pain management, and their responses are particularly consequential for TGD youth (98). Supportive, affirming caregivers can buffer minority stress, facilitate access to gender-affirming and pain care, and reinforce adaptive coping and activity engagement. In contrast, non-affirming or rejecting caregivers may contribute to identity concealment, loss of social support, worse mental health, and reduced access to both gender-affirming and pain services (67). Many TGD youth are not “out” to all caregivers, school personnel, or medical providers, which can limit their ability to disclose both identity-related stress and pain. Navigating decisions about disclosure in the context of chronic pain (e.g., whether to share gender identity with a pain specialist, or pain with a gender clinic) may itself be a source of stress and can shape adherence, trust, and treatment trajectories.

#### Identity and development

Adolescence is a key developmental period for both identity formation and the onset of many chronic pain conditions (68, 69). For TGD youth, processes of gender identity development, exploration, and affirmation unfold alongside the challenges of living with chronic pain. Pain-related disability (e.g., school absence, reduced social participation) may interfere with opportunities for gender exploration, community connection, and access to affirming spaces, whereas gender dysphoria and identity-related stress may intensify bodily vigilance or dissociation and pain-related distress (70). Considering identity development as a dynamic context for pain—rather than a static background characteristic—may help clinicians and researchers better understand trajectories of both gender affirmation and chronic pain across adolescence.

#### An intersectional lens

An intersectional lens is essential for understanding chronic pain in TGD youth. Gender identity does not exist in isolation; TGD youth also navigate systems shaped by race, ethnicity, culture, disability, socioeconomic status, and religious or spiritual context, all of which can accumulate and lead to chronic or traumatic stress experienced by transgender youth (65). TGD youth of color, for example, may experience racism, transphobia, and pain-related stigma simultaneously, with cumulative effects on stress physiology, access to affirming care, and trust in medical systems. Likewise, youth from highly religious or culturally conservative communities may face heightened concealment demands or family conflict, while TGD youth with neurodevelopmental disabilities may encounter additional communication and accessibility barriers. Applying an intersectional framework highlights that chronic pain risk is shaped not only by individual identities, but also by structural forces that distribute stress and resources unevenly across groups.

TGD youth with chronic pain may experience “double stigma”: marginalization related to gender identity and expression, as well as skepticism or stigmatizing attitudes related to chronic pain (e.g., being perceived as exaggerating or “faking”) (11). Experiences of being disbelieved, blamed, or minimized—whether in relation to pain, gender, or both—can compound feelings of social isolation, hopelessness, and mistrust of healthcare providers. These compounding stigmas likely intensify minority stress processes and may further sensitize stress and pain systems over time.

### Societal and systemic factors

Gender-affirming care includes social, psychological, legal, and medical approaches that support and affirm gender identity (71). At the societal and systems level, access to gender affirmation has been shown to improve overall quality of life and resilience (72) and disrupt the root causes of psychosocial distress (73, 74), potentially mitigating or preventing downstream effects on chronic pain and its associated disability. A number of studies have shown that gender-affirming hormone therapy (GAHT) leads to reductions in anxiety, depression, and suicidality in TGD youth overall and in comparison to TGD youth without hormone therapy (62, 75–78). Longitudinal data from a 2-year prospective cohort study further indicates that initiation of GAHT is associated with sustained improvements in appearance congruence, positive affect, and life satisfaction (79). By alleviating the psychological distress associated with gender dysphoria and societal stigma, access to gender-affirming care may buffer against the physiological and psychological processes that exacerbate chronic pain.

Everyday gender-affirming practices can also intersect with pain experiences. For example, chest binding may substantially reduce dysphoria and improve safety and participation in social situations, but it can also contribute to musculoskeletal discomfort, chest or back pain, shortness of breath, and skin irritation (10, 80). Similarly, tucking, packing, or intensive voice training may lead to localized pain or strain (99). These practices should not be framed as inherently pathological; rather, clinicians can adopt a harm-reduction approach—routinely and non-judgmentally asking about gender-affirming strategies, collaboratively discussing risks and warning signs, and offering practical guidance (e.g., use of safe products, optimizing fit, limiting hours of binding/tucking, encouraging rest days and good hygiene) to help youth balance gender affirmation and bodily comfort.

Despite evidence for the benefits of gender-affirming care, societal discrimination and stigma of TGD identities significantly hinders access and participation in social systems, including healthcare, education, and employment, leading to systemic health disparities (81). In healthcare settings, TGD youth often face provider discrimination, inadequate or harmful care, dismissal, or denial of services (81–83). For TGD individuals with chronic pain, systemic barriers prevent transgender youth from accessing appropriate pain management interventions, leaving them without effective strategies to address their chronic pain (9, 10, 84). These healthcare disparities underscore the urgent need for inclusive policies and provider training to ensure equitable access to care.

Legal and policy-related stressors add a notable dimension to the societal challenges faced by transgender youth (85, 86). Policies vary considerably in the U.S., reflecting a “patchwork” of inclusionary and exclusionary policies, many of which criminalize gender-affirming care and protections (87). In states/regions where transgender rights are more severely restricted, youth experience chronic fear, anxiety, and increased suicidality (88), especially without access to gender-affirming care or abrupt discontinuation of access due to the rapidly evolving legal landscape (89). Policies that restrict access to gender-affirming care, bathrooms, sports participation, or educational opportunities reinforce systemic discrimination and minority stress (85), very likely worsening psychosocial wellbeing and exacerbating physiological mechanisms relevant to pain processing for the TGD individual. Advocacy for inclusive policies and legal protections is critical for reducing the societal burden on TGD youth and mitigating the health consequences of chronic stress.

## Discussion

This review of existing literature highlights that TGD youth face a convergence of neurobiological vulnerabilities, psychosocial distress, and social and societal determinants of health that overlap with known risk factors for pediatric chronic pain. Chronic pain in TGD youth is likely driven by a complex interplay of central sensitization, hormonal influences, minority stress, and systemic barriers, which together create a uniquely challenging pain experience. Despite these insights, substantial gaps remain in our understanding of how gender identity intersects with pain processing and biopsychosocial influences. Addressing these gaps will require methodologically rigorous studies that integrate diverse research modalities, including neuroimaging, biomarker analyses, and patient-reported outcomes—situated in the context of legal and policy-related stressors—to capture a holistic picture of pain trajectories in this population. Longitudinal studies, in particular, are essential to understanding the development and persistence of chronic pain in TGD youth over time and the impact of interventions aimed at mitigating this burden.

One critical area for future research is the role of gender-affirming medical interventions in pain perception and management. While existing research on the impact of sex hormones on chronic pain may suggest that hormone therapy may influence pain sensitivity—potentially through estrogen's pro-nociceptive effects or testosterone's anti-nociceptive properties (28, 29)—the long-term effects of these treatments on chronic pain outcomes remain unclear. Moreover, the timing of interventions, such as the use of puberty blockers or the initiation of GAHT, may play a significant role in shaping pain experiences. For example, delaying puberty with gonadotropin-releasing hormone (GnRH) agonists (“puberty blockers”) may theoretically influence neurodevelopmental processes involved in pain modulation, given the role of sex hormones in shaping nociceptive and affective circuits. However, empirical evidence regarding the long-term neurodevelopmental and pain-related outcomes of puberty suppression in TGD youth remains limited (30), and no studies to our knowledge have directly examined chronic pain endpoints (48). Careful, longitudinal, and mechanistic research is needed to clarify whether and how pubertal suppression and subsequent GAHT affect pain trajectories in this population. Understanding how medical interventions interact with pain processing in TGD youth is critical for refining clinical guidelines and ensuring that gender-affirming care is optimized to address their unique health needs. Research in this area should also account for individual differences, such as the presence of co-occurring conditions like autism or mental health conditions, which may further influence pain outcomes.

Future research in this area should also adopt participatory and equity-oriented approaches (90). Including TGD youth with lived experience of chronic pain as patient partners and co-researchers—across study design, measure selection, interpretation of findings, and dissemination—can help ensure that research questions are relevant, measures are acceptable, and interpretations honor community priorities. Such partnerships are also consistent with broader efforts to reduce power imbalances in research and to promote health equity for TGD youth (91, 92).

Beyond research, systemic changes in healthcare delivery and policy are essential for addressing chronic pain disparities in TGD youth. Significant systemic barriers pertaining to healthcare access, including lack of insurance coverage, provider bias, and threats or actual criminalization of healthcare providers providing gender-affirming care (100–102) are just a few examples of systemic barriers that limit general healthcare for TGD youth, let alone intervention for chronic pain. To mitigate these disparities, healthcare systems must prioritize training medical professionals in LGBTQ+-affirming care, equipping providers with the knowledge and skills to recognize and address the pain-related needs of TGD patients (84). Additionally, policies should ensure equitable access to gender-affirming treatments, mental health services, and pain management interventions.

Broader societal interventions are also crucial for addressing the upstream determinants of chronic pain in this population. Anti-discrimination laws, inclusive school policies, and community-based support systems are vital for reducing the minority stress that amplifies pain vulnerability. For example, school-based programs that foster acceptance and provide gender-affirming resources can reduce bullying and victimization, which are significant contributors to both psychological distress (61, 93) and physical pain. Similarly, community programs that provide safe spaces, peer support, and access to affirming resources can buffer against the negative effects of family rejection and social isolation. Advocacy efforts must also address legal and policy-related stressors, such as restrictions on access to bathrooms, sports participation, or healthcare, which perpetuate systemic discrimination and contribute to chronic stress and its associated health consequences.

In conclusion, chronic pain in TGD youth is a multifaceted and understudied issue that demands interdisciplinary and systemic approaches for effective intervention. Addressing this complex health disparity requires advancing research to explore the biopsychosocial mechanisms underlying pain, improving clinical practices to provide inclusive and patient-centered care, and advocating for policies that promote equity and reduce minority stress. By prioritizing these efforts, we can work toward mitigating chronic pain disparities and improving the overall quality of life for TGD youth. Future initiatives should emphasize inclusivity, biopsychosocial care acknowledging the biological, psychological, and social factors contributing to pain, and trauma-informed approaches to address the determinants of pain in this population, with close collaboration between pediatric pain teams, gender-affirming care providers, and TGD youth and families themselves.
